# Nutrient Patterns and Risk of Osteopenia in Postmenopausal Women

**DOI:** 10.3390/nu15071670

**Published:** 2023-03-29

**Authors:** Patricia Paula da Fonseca Grili, Camila Vilarinho Vidigal, Geise Ferreira da Cruz, Ben-Hur Albergaria, José Luiz Marques-Rocha, Taísa Sabrina Silva Pereira, Valdete Regina Guandalini

**Affiliations:** 1 Postgraduate Program in Nutrition and Health, Health Sciences Center, Federal University of Espirito Santo, Vitória 29047-105, Brazil; 2 Department of Integrated Education, Health Sciences Center, Federal University of Espirito Santo, Vitória 29047-105, Brazil; 3 Department of Social Medicine, Federal University of Espirito Santo, Vitória 29047-105, Brazil; 4 Department of Health Science, University of the Americas Puebla, Puebla 72810, Mexico

**Keywords:** bone mineral density, bone health, osteoporosis, menopause, nutrient patterns

## Abstract

Nutrient patterns (NPs) and the synergistic effect between nutrients have been shown to be associated with changes in bone mineral density (BMD). This study aimed to identify NPs and to associate them with BMD categories in postmenopausal women. This cross-sectional, observational, analytical study was carried out with women in menopause for at least 12 months, aged ≥50 years. Sociodemographic, lifestyle, and clinical variables were investigated. BMD was assessed using dual energy X-ray absorptiometry. A dietary assessment was conducted using a food frequency questionnaire, and three nutrient patterns (NP1, NP2, and NP3) were extracted from the principal component analysis. Multivariate logistic regression was applied to investigate the association between BMD classifications and NP consumption. A total of 124 women, aged on average, 66.8 ± 6.1 years, were evaluated. Of these, 41.9% had osteopenia and 36.3% had osteoporosis. The NP1 (OR: 6.64, [CI95%: 1.56–28.16]; *p* = 0.010), characterized by vitamin B12, pantothenic acid, phosphorus, riboflavin, protein (total and animal), vitamin B6, potassium, vitamin D, vitamin E, calcium, cholesterol, β-carotene, omega 3, magnesium, zinc, niacin, and selenium; and the NP2 (OR: 5.03, [CI95%: 1.25–20.32]; *p* = 0.023), characterized by iron, vegetable protein, thiamine, folate, fibers (soluble and insoluble), PUFA, vitamin A, vitamin K, alpha-tocopherol, copper, sodium, and retinol, was inversely associated with osteopenia. The lower consumption of NP1 and NP2 by postmenopausal women was associated with a higher risk of osteopenia, but not osteoporosis.

## 1. Introduction

Skeletal disorders such as osteopenia and osteoporosis result from reduced bone mineral density (BMD) and the deterioration of bone microarchitecture [1,2]. Osteoporosis and osteopenia affect about 24.8% and 39.4% of women in general, respectively [3]. In post-menopausal women, the prevalence is higher, reaching 27.4% for osteoporosis and 42.1% for osteopenia [3]. This scenario is mainly due to the hypoestrogenism characteristic of menopause, which increases bone resorption and decreases bone formation [4,5]. Although inherent to the course of life and aging, these changes increase the risk of falls and fractures, worsen the quality of life, and can make women more dependent. Furthermore, they can generate high costs with medicines, surgeries, and hospitalization [6,7].

In addition to hormonal changes, several modifiable factors such as smoking, physical activity, and dietary factors are associated with BMD in postmenopausal women and with the possible deleterious outcomes in the bone health of this population [5,8,9]. Some nutrients such as calcium, vitamin D, and proteins are known to act on bone structure and metabolism, consequently preventing skeletal disorders [10]. However, in a diet, foods and consequently, the nutrients that they contain, are consumed together rather than independently. Therefore, evaluating the effect of a dietary pattern and not just that of an isolated nutrient may be more advantageous [11,12]. Nutrient patterns (NPs) are an alternative way to examine the relationship between diet and chronic diseases. This approach aggregates food constituents based on the degree to which they correlate with each other. This allows for the assessment of interactions, cumulative, and synergistic effects, not to mention changes in the bioavailability of nutrients that can be correlated to health outcomes, which would not be possible when these nutrients are considered alone [12]. Evaluating nutrients in a consumption pattern may offer a different perspective for the prevention and treatment of chronic diseases. Different methods are available for characterizing NPs based on the assessment of dietary intake (a priori and a posteriori methods) [11,12].

The relationship between NPs and BMD in older adults, especially postmenopausal women, has only recently received attention [13,14,15,16]. In a study with the elderly, a mixed NP, with a high consumption of nutrients from animal and plant sources, including protein, calcium, and vitamin B12, among others, was positively associated with BMD, while nutrient patterns from animal sources and plant sources did not show an association [14]. On the other hand, a prospective study with a 20-year follow-up found that a nutrient pattern rich in foods of animal origin was associated with a higher risk of fractures in the elderly [17]. A study of 151 Iranian postmenopausal women showed a positive association between an NP rich in folate, fiber, vitamin B6, potassium, vitamin A, vitamin C, β-carotene, vitamin K, magnesium, copper, and manganese, and the BMD of lumbar spine [13]. However, an NP with nutrients recognized as being essential for bone health, such as calcium, vitamin D, and proteins, was not associated with BMD [13]. Ilesanmi-Oyelere et al. [15] evaluated 101 Australian postmenopausal women and observed that an NP rich in riboflavin, calcium, phosphorus, potassium, and proteins was positively associated with BMD of the lumbar spine and femoral neck [15]. To date, only these studies have characterized NPs in postmenopausal women and have associated them with BMD, having shown different nutrient consumption patterns and heterogeneous results regarding their effects at different BMD sites.

Considering the heterogeneity of the results of previous studies and the lack of a conclusive NP to address BMD, it is necessary to clarify which patterns may be related to bone mass and the consequent development of disorders such as osteopenia and osteoporosis. Furthermore, by evaluating the consumption patterns of postmenopausal women and their correlation with the aforementioned outcomes, it may be possible to add new nutritional recommendations for the prevention and treatment of osteoporosis. In this sense, the aim of this study was to identify NPs and to associate them with the BMD categories of postmenopausal women.

## 2. Materials and Methods

### 2.1. Study Design, Sample Size, and Population

**Figure 1:**
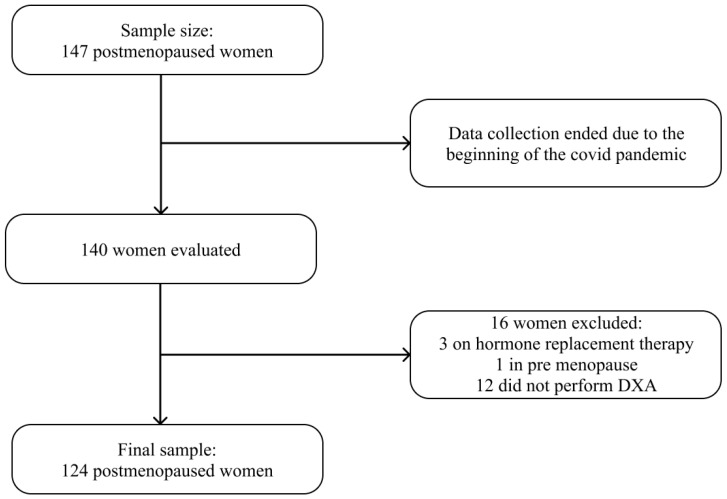
Sample selection flowchart.

This observational, cross-sectional, analytical study of probability sampling was conducted in a climacteric and osteoporosis outpatient clinic of a university hospital in Vitória, Espírito Santo, Brazil, between June 2019 and March 2020. For sample calculation, we used the Open-Source Epidemiologic Statistics for Public Health (OpenEpi^®^ Version 3.01, Atlanta, GA, USA) software [18]. A confidence interval of 95%, a margin of error of 5%, and a prevalence of osteoporosis in women over 50 years of 21.3% were considered [19], resulting in a sample of 147 women. Sample calculation and size have already been detailed in a previous study [20]. In this study, women aged ≥50 years and in menopause for at least 12 months were included, while those under hormone replacement therapy (HRT) and who did not have dual energy X-ray absorptiometry (DXA) results in their medical records were excluded. Due to the onset of the COVID-19 pandemic, collections were terminated ahead of schedule, and the predicted sample size of 147 women was not reached. A total of 140 women were evaluated. Among these, one was excluded for being premenopausal, three for being under HRT, and 12 for not having DXA data in their medical records, resulting in 124 women in the final sample (Figure 1).

### 2.2. Outcome Variable

The main outcome of this study was the presence of osteopenia and osteoporosis assessed from BMD, obtained using DXA (GE Lunar Prodigy Advance^®^, GE Healthcare, Chicago, IL, USA), duly calibrated, and using the GE Encore^®^ software, version 14.10, configured to use the National Health and Nutrition Examination Survey [21]. The BMD of the femoral neck and lumbar spine (L3 and L4 positions) was evaluated. All densitometry tests were performed by a trained radiology technician and the result reported by a single specialist physician to avoid interobserver variation. Data were extracted from medical records, and the results of exams performed up to six months before or after the study were included. Participants were classified into three groups: (1) Normal BMD (T-score ≥ −1.0 SD), (2) Osteopenia (T-score between −1.0 and −2.5 SD), and (3) Osteoporosis (T-score ≤ −2.5 SD) [22].

### 2.3. Exposure Variable

To assess food consumption, we applied the reduced version of the ELSA-Brasil Food Frequency Questionnaire (FFQ), a semi-quantitative FFQ adapted and validated for the Brazilian population [23,24]. The questionnaire contains 76 food items and is structured in 3 sections: (1) food/preparations; (2) measurements of consumption portions; and (3) consumption frequency, with 8 response options: “more than 3 times/day”, “2–3 times/day”, “1 time/day”, “5–6 times/week”, “2–4 times/week”, “1 time/week”, “1–3 times/month”, and “never/almost never” [23]. Information was collected on the frequency and amount by which the participants consumed food in the last 12 months. For a better understanding, a response card with the frequencies and a kit of requirements with home measures was made available [23].

The nutrients present in the FFQ foods were quantified using the Nutrition Data System for Research^®^ (NDSR) software [25]; further descriptions of the composition table used have already been described previously [23,24]. After extracting the nutritional composition data from the FFQ, the total energy consumption was adjusted through the residual method [26] using the mean energy of each BMD group (normal, osteopenia, and osteoporosis). The plausibility of the energy intake data was verified and evaluated using the Goldberg cut-off point [27,28]. More details about the plausibility can be seen in a previous publication [20]. The average EI/BMR ratio was 1.61. A total of 64.5% (*n* = 80) of the women had energy consumption within the limits defined, 12.9% (*n* = 16) had underreported energy consumption, and 22.6% (*n* = 28) had overreported energy consumption, making it plausible to use all of the FFQs in the analyses.

#### Nutrient Patterns (NPs)

To determine NPs, factor analysis (FA) was performed through principal component analysis (PCA) [11]. Before extracting the patterns (factors), the correlation matrix of the 39 nutrients was examined to justify the choice of factors. Bartlett’s sphericity test was significant (*p* < 0.001), and the Kaiser-Mayer-Olkin (KMO) measure of sampling adequacy had a score of 0.661, indicating that a correlation between the variables was adequate for the analysis [29]. Three main NPs were extracted for the analysis according to the following criteria: factors with eigenvalues >1.0, the identification of an inflection point in the scree plot, and the natural interpretation of the factor [29,30]. The varimax rotation was applied to obtain a simpler matrix to interpret, finding the factor loading of each nutrient [30]. The factor loading is the correlation coefficient between the nutrients in each of the identified patterns. Nutrients whose factor loadings were >0.30 or ≤−0.30 had a strong relationship with the pattern; the greater the load, the greater the relationship between a given nutrient and a factor, while the plus and minus signs refer to direct associations and the lack thereof, respectively [30]. After identifying the factors, each individual received a score for each nutrient pattern, and this score was obtained by summing the intake of each nutrient weighted by the factor loading [30]. Finally, the individuals had their scores divided into tertiles (*n* = 41, in the first and second tertiles; *n* = 40, in the third tertile). The first and second tertiles represent the lowest consumption of that pattern, while the third tertile represents the highest consumption.

### 2.4. Covariates

Sociodemographic data such as age (years) were collected. Self-declared color [31] was later classified as “white” and “non-white”; education level was categorized as “no schooling”, “elementary school”, “high school”, and “university education”; marital status was categorized as “with a partner” and “without a partner”; and employment status was categorized as “employed” and “unemployed”. Regarding lifestyle habits, we evaluated alcohol consumption (“consume”, “do not consume”), smoking (“smoker”, “non-smoker”), and physical activity (PA) level, which were obtained from the International Physical Activity Questionnaire (IPAQ) [32]. To avoid overestimating the PA level, only the sum of issues related to leisure and transportation was considered. Women who reported performing at least 150 min of PA per week were classified as “Sufficiently active”, while those who reported doing less than 150 min of PA weekly were classified as “Insufficiently active”, using the World Health Organization (WHO) recommendation [33].

Clinical data regarding the time since menopause were both self-reported and obtained from the participant’s current age minus the age at which menopause was established and presented in years. The uses of calcium and vitamin D supplementation, and of antiresorptive drugs, were collected from the medical records and categorized into “uses” and “does not use”. To assess the nutritional status, height (m) and body mass (kg) were collected, as previously reported [34]. From these variables, the body mass index (BMI) (kg/m^2^) was calculated by dividing body mass by height squared [34]. Women up to 59 years of age were classified according to the WHO [35], while women aged ≥60 years were classified according to the Pan American Health Organization (PAHO) [36].

### 2.5. Ethical Aspects

Individuals participated voluntarily and provided written consent by signing the Free and Informed Consent Term, after having had the research read and clarified to them so that they were aware of the study, guaranteeing their anonymity and the confidentiality of the information obtained. The study was conducted in accordance with the Declaration of Helsinki, and was approved by the Research Ethics Committee of Health Sciences Center of the Federal University of Espírito Santo, under protocol number: 2,621,794.

### 2.6. Statistical Analysis

The sample was characterized through the distribution of frequencies and the estimation of measures of central tendency and dispersion. The normality of the variables was assessed using the Kolmogorov-Smirnov test. One-way ANOVA and Kruskal-Wallis tests were applied to verify the difference between means according to data normality, while the chi-squared and Fisher’s Exact tests were applied to verify the difference between proportions. The Tukey and Bonferroni post hoc tests were applied to assess statistical differences between groups of parametric and non-parametric variables, respectively.

Each nutrient pattern was classified according to tertiles of consumption. The odds ratios (OR) and their respective confidence intervals (CI) were calculated, taking the third tertile of the NP as a reference, and osteopenia and osteoporosis as the outcome. For the multivariate analysis, three adjustment models were made: the first model adjusted for age and time since menopause, and BMI; the second model added the level of physical activity and antiresorptive drugs to model 1; and the third model added to model 2 the use of calcium and vitamin D supplements. Data were analyzed using SPSS^®^ software version 22.0 and the significance level adopted for all tests was 5.0%.

## 3. Results

The women in this study had a mean age of 66.8 ± 6.1 years, the mean time since menopause was 19.6 ± 8.8 years, and the mean BMI was 27.3 ± 4.7 kg/m^2^. According to the BMD categories, 21.8% of the participants had normal BMD, 41.9% had osteopenia, and 36.3% had osteoporosis. Women with osteopenia and osteoporosis had more time since menopause (*p* = 0.002), and they were between 60.0 and 69.9 years old (*p* = 0.022). Women with osteoporosis were the oldest (*p* = 0.010) and the ones with the lowest BMI (*p* < 0.001) (Table 1). The other variables were not significantly different in the BMD groups.

Regarding nutritional status, 40.7% of women with normal BMD were classified as obese, while 44.2% of women with osteopenia and 46.7% of women with osteoporosis were eutrophic (*p* < 0.001). When evaluating the clinical data, we observed that among the women who used calcium supplementation, 43.2% had osteopenia and 45.7% had osteoporosis (*p* < 0.001). Of those who received vitamin D supplementation, 43.7% had osteopenia and 46.5% had osteoporosis (*p* < 0.001). Regarding the use of antiresorptive drugs, 60.3% of the women who used them had osteoporosis (*p* < 0.001) (Table 1).

The mean energy consumption was 2013.1 ± 791.4 kcal/day and there was no significant difference among the BMD categories (*p* = 0.357) (Table 2). Women with osteopenia and osteoporosis had a lower mean consumption of total lipids, saturated fat, polyunsaturated fatty acids (PUFA), monounsaturated fatty acids (MUFA), selenium, and omega 3 when compared with women with normal BMD (*p* < 0.05 for all). The osteopenia group consumed lower amounts of carbohydrates, riboflavin, folate, calcium, zinc, phosphorus, and potassium compared with the normal and osteoporosis group, and they consumed less proteins, vegetable protein, alpha tocopherol, vitamin E, pantothenic acid, vitamin B12, iron, and sodium, when compared with the normal group. Thiamine consumption was different in the three groups, with the osteoporosis group showing the lowest consumption of this nutrient (*p* < 0.05). As for the other nutrients, there was no significant difference between groups (Table 2).

Three NPs were extracted from the PCA, explaining 56.7% of the total variance in nutrient intake (Table 3). Nutrient pattern 1 (NP1) explained 21.9% of the nutrient intake variance, and was characterized by the highest consumption of vitamin B12, pantothenic acid, phosphorus, riboflavin, animal protein, total protein, vitamin B6, potassium, vitamin D, vitamin E, calcium, cholesterol, β-carotene, omega 3, magnesium, zinc, niacin, and selenium. Nutrient pattern 2 (NP2) explained 20.4% of the variance and was characterized by the highest consumption of iron, vegetable protein, thiamine, folate, total fiber, PUFA, insoluble fiber, vitamin A, vitamin K, alpha-tocopherol, copper, sodium, and retinol. Finally, nutrient pattern 3 (NP3) was characterized by a high consumption of carbohydrates, total sugar, soluble fiber, and vitamin C; and a low consumption of total lipids, MUFA, saturated fats, and trans-fat, explaining only 14.4% of the variance.

When analyzing the tertiles of consumption of each nutrient pattern, there was a significant difference between the BMD classifications regarding NP1 (*p* = 0.038). While 70.9% of women with osteopenia were in the first and second tertiles of NP1 consumption, 55.6% of women with normal BMD were in the third tertile of NP1 consumption. Regarding women with osteoporosis, an equal distribution was observed among the tertiles of NP1. In women showing a higher consumption of NP3, there was a significant difference among the different levels of physical activity (*p* = 0.005). There was a higher proportion of insufficiently active women in the first and second tertiles of NP3 (Table S1).

Table 4 presents the association between the tertiles of NP1 and NP2, and the BMD categories based on the multivariate logistic regression. Although NP2 was not significantly different among the BMD categories (*p* = 0.053) in the bivariate analyses, when included in the regression models, this pattern was associated with osteopenia. In the crude model, women who were in the first tertile of NP1 were more likely to be diagnosed with osteopenia (OR: 6.00 [95%CI: 1.73–20.82]; *p* = 0.005) when compared with women in the third tertile of consumption. The same was observed in the second tertile of NP1 (OR: 3.70 [95%CI: 1.15–11.86]; *p* = 0.028). After adjustments for possibly confounding variables, women in the first and second tertiles still showed a risk of developing osteopenia when compared with those in the third tertile. In fact, this risk increased among women in the first tertile of NP1 (OR: 6.64, [95%CI: 1.56–28.16]; *p* = 0.010), which was also true for those in the second tertile (OR: 5.15, [95%CI: 1.32–20.07]; *p* = 0.018) (Table 4).

When evaluating NP2, in the crude model, women in the first and second tertiles had a higher risk of being diagnosed with osteopenia (OR: 4.84, [95%CI: 1.37–17.09]; *p* = 0.014; OR: 3.50, [95%CI: 1.13–10.84]; *p* = 0.030, respectively) when compared with women in the third tertile. In the adjusted models, the first tertile of NP2 consumption remained associated with osteopenia, with an increased risk (OR: 5.03, [95%CI: 1.25–20.32]; *p* = 0.023), while the second tertile lost its association with osteopenia (OR: 3.59, [95%CI: 0.98–13.13]; *p* = 0.054) when adjusting for PA level, antiresorptive drugs, and calcium and vitamin D supplementation. We found no association between the NPs and osteoporosis in the model presented (*p* > 0.05) (Table 4).

## 4. Discussion

In the present study, NP1 (characterized by the consumption of vitamin B12, pantothenic acid, phosphorus, riboflavin, animal protein, total protein, vitamin B6, potassium, vitamin D, vitamin E, calcium, cholesterol, β-carotene, omega 3, magnesium, zinc, niacin, and selenium) was inversely associated with osteopenia. In other words, the lower consumption of this pattern increases the risk of postmenopausal women of having compromised BMD. This same association was observed for NP2, defined by a high consumption of iron, vegetable protein, thiamine, folate, total fiber, PUFA, insoluble fiber, vitamin A, vitamin K, alpha-tocopherol, copper, sodium, and retinol.

The NP1 and NP2 identified in our study are composed of a large number of nutrients that are important for bone health; hence, their association with osteopenia. Bone is composed mainly of proteins, which, in addition to playing a structural role, have an anabolic effect via insulin-like growth factor 1 (IGF-1) [10]. IGF-1 also acts on calcium and phosphorus absorption in the intestine, and phosphate reabsorption by the kidney [10]. Calcium is a component of, and gives rigidity to bones, when incorporated into collagen fibers in the form of hydroxyapatite [10,37,38]. Other key nutrients are: vitamin D, which is responsible for calcium homeostasis; potassium and magnesium, which participate in calcium regulation; and vitamin K, which is involved in the formation of the bone matrix during mineralization [10,37,38]. Furthermore, omega-3 and PUFAS can inhibit osteoclast activity, and consequently, bone resorption, in addition to stimulating bone formation [37,39]. In postmenopausal women, omega-3 has been shown to have a slight effect in decreasing bone turnover [40]. However, the results are controversial, as the consumption of PUFAS either did not affect bone health or it had negative effects, such as a small increase in the risk of fractures in women [41,42]. In a study with elderly women, the consumption of PUFAS was positively associated with the lumbar spine in women who were not using RHT [43]. The possible role of PUFAS in bone health can be explained by the regulation of cell maturation and activity, since they act on mechanisms such as prostaglandins, which are present in bones and which are derived from PUFAS; can increase bone marrow cell numbers and influence osteoblastic differentiation; altering the RANKL/OPG ratio and decreasing osteoclastogenesis [44]. The conflicting results found in the studies may be due to several factors, such as the different methodologies applied in the way of measuring dietary intake, the outcomes and exposures evaluated, the type of population studied, and factors such as the dietary and life profile of the population; therefore, more population studies with better methodologies should be performed. B-complex vitamins, which act as coenzymes for energy generation, were also correlated with the patterns found, although their role in bone health is still inconsistent, especially regarding thiamine (B1), riboflavin (B2), niacin (B3) [45], and pantothenic acid [B5]. In rodents, these vitamins influence osteoclastogenesis through the suppression of reactive oxygen species (ROS) [46], although this hypothesis has not yet been confirmed in humans.

The role of other B-complex vitamins in bone health is still unclear, but there are indications that they are linked to homocysteine metabolism, which involves remethylation that is dependent on vitamin B12 and folic acid (B9), and trans-sulfuration that is dependent on vitamin B6 [47]. Evidence obtained in vitro and in animal models suggests that high plasma homocysteine concentrations, driven by low concentrations of vitamins B12 and B9, increase osteoclast activity and bone resorption, reducing bone strength [47,48]. Furthermore, this can lead to an increase in free radicals and oxidative stress, with consequent endothelial dysfunction and lower blood flow to the bone, which results in lower nutrient availability and micro-damage to the bone tissue [47]. Observational studies found an association between lower consumption and the plasma levels of vitamin B6, with lower BMD and a higher risk of fractures in elderly men and postmenopausal women [49,50,51]. Regarding folate, the results concerning its association with BMD in postmenopausal women are still divergent [50,52,53,54]. Likewise, the reports on vitamin B12 are also heterogeneous [45,52,55,56].

Among minerals, zinc and copper are essential cofactors for enzymes involved in bone matrix synthesis, acting to stimulate bone formation and to suppress its remodeling [57], with positive results regarding BMD [58,59,60,61]. Oxidative stress seems to have an influence on BMD, since ROS and free radicals are involved in osteoclastogenesis and osteoblast apoptosis [62], and nutrients such as selenium, β-carotene, vitamin E, alpha-tocopherol, and copper have antioxidant properties and may be important for bone protection against oxidative stress [58,63,64].

Furthermore, fibers seem to increase intestinal calcium absorption in both rats and humans, and improve bone parameters [65,66,67]. In a randomized cross-over trial with postmenopausal women, the consumption of soluble corn fibers increased bone calcium retention, improving bone calcium balance [66]. In rats, soluble fibers increased whole-body bone mineral content and femoral BMD, and resistance to fracture [67]. Fructooligosaccharides (FOS) increased calcium and magnesium absorption, and increased bone mineralization in rats [68]. Soluble fiber seems to demonstrate more benefits for bone health than insoluble fiber. The greater role of soluble fiber in bone health may be due to a greater degree of fermentation and viscosity [69]. Despite this, in our study, only total fiber and insoluble fiber (NP2) were associated with BMD. These discrepancies emphasize that more population studies in humans should be performed to better unravel the role of fibers in bone health.

NP3, characterized by a high consumption of carbohydrates, soluble fiber, total sugar, and vitamin C, while being low in total lipids, saturated fat, and MUFA, was not associated with osteopenia and osteoporosis. That may be due to the low number of nutrients correlated in this pattern, in addition to the absence of important nutrients for bone health [9], although vitamin C is a potent antioxidant that is associated with BMD [63]. As this pattern explains only 14.4% of the variance of the factors, this low correlation may have influenced the lack of association between NP3 and the outcomes investigated.

In contrast to our initial hypothesis, no association was observed between NP and osteoporosis. The explanation for the lack of association may lie in the characteristics of the population studied. Of the 124 women evaluated, only 45 were classified as having osteoporosis, and their distribution between NP1 and NP2 was uniform, which may have caused the loss of effect on BMD. In addition, because of the characteristics of the study, this condition was previously established, and the women may have modified their consumption patterns because they were oriented on the importance of calcium, vitamin D, and other nutrients for bone health during outpatient treatment.

Our results are consistent with those from the few other studies available on the relationship between NP and BMD in postmenopausal women [13,15]. In a study by Karamati et al. [13] with 151 postmenopausal Iranian women, an NP rich in folate, total fiber, vitamin B6, potassium, vitamin A, vitamin C, β-carotene, vitamin K, magnesium, copper, and manganese was positively associated with lumbar spine BMD, but not with that of the femoral neck in this population, while an NP rich in vitamin B2, protein, calcium, phosphorus, zinc, vitamin B12, and vitamin D; and low in vitamin E, nutrients commonly known for their action on bone metabolism, was not associated with any site of BMD [13]. Ilesanmi-Oyelere et al. [15], when evaluating 101 post-menopausal Australian women, found a positive association between an NP rich in riboflavin, phosphorus, calcium, sugar, potassium, vitamin B6, carbohydrate, magnesium, thiamine, sodium, iron, iodine, niacin, and vitamin B12, and the lumbar spine and femoral neck BMD; while an NP rich in PUFA, alpha-tocopherol, linoleic acid, alpha-carotene, eicosapentaenoic acid (EPA), and docosahexaenoic acid (DHA) was negatively associated with hip BMD but lost association when adjusting for age, BMI, and physical activity [15]. In addition, Melaku et al. [14], who evaluated 1135 elderly Chinese, observed an increase of one unit in the Z score of a mixed NP, characterized by a high consumption of nutrients such as phosphorus, potassium, calcium, niacin, starch, and dextrin; vitamins B1, B2, B3, B7, and B12; fiber, protein, and retinol, which was associated with a 9.5 mg/cm^2^ increase in BMD [14]. It is noted that because different NPs naturally differ from each other, it is not possible to indicate which pattern would be best for maintaining BMD. However, the present study and the aforementioned ones demonstrate that balanced diets based on vegetables, oilseeds, fruits, fish and milk, and dairy products may be beneficial for maintaining bone health and for preventing osteoporosis [16]. Furthermore, it is worth noting that some nutrients, in addition to calcium and vitamin D, such as vitamin A, B12, riboflavin, and niacin, are present in several NPs and should be evaluated in future studies for a better understanding of their roles in bone health.

This study has some limitations, such as the cross-sectional design that does not allow us to infer the causality between the NP identified and osteopenia. There is a need for longitudinal studies, especially in this population, in order to clarify the relationship between nutrients in the development of osteopenia and osteoporosis. The use of drugs such as glucocorticoids, and other chronic diseases such as cancer, diabetes, chronic kidney diseases, and human immunodeficiency virus (HIV), which may increase the risk of osteoporosis, were not evaluated, which could be a confounding factor [70]. The measurement of physical activity was also a limitation, since due to the tool being used, it was not possible to measure the type of physical activity performed by these women. Strength and endurance exercises can affect bone health in different ways [71,72,73]. Actual evidences indicate that a combination of the two types of exercise has small but positive effects on bone health and osteoporosis prevention [73,74]. Women using antiresorptive drugs were not excluded from the analyses, although this parameter was used with adjustment in the multivariate model. In addition, the quantification of nutrients from the FFQ may have been hampered by the inherent flaws of this method in assessing food consumption [75], although food consumption was assessed using a validated questionnaire for the population studied [23,24]. Among the strengths of the present study, we highlight obtaining BMD via DXA of the lumbar spine (L2–L4) and femoral neck, the gold standard method for the diagnosis of osteoporosis [22]. In addition, women under HRT were excluded, and by adjusting the multivariate model for potential confounders, the reliability of the results was strengthened. Furthermore, the present study has some implications for clinical practice. The results indicate that the adoption of an NP rich in nutrients such as vitamin B12, calcium, selenium, proteins, fibers, and others throughout life can modulate the degree of bone deterioration, reducing the chances of presenting osteopenia/osteoporosis in the postmenopausal period. In this context, dietary adjustments should be made, especially for those with low weight, endocrine disorders, chronic diseases, and other risk factors. It is worth noting that this study innovates by studying dietary patterns based on PCA, meeting the strict criteria of assumptions required for the implementation of this method. Last but not least, studies that analyzed the dietary patterns of postmenopausal women are rare.

In conclusion, a lower consumption of NP1 and NP2 was negatively associated with BMD. So, postmenopausal women in this study were at greater risk of osteopenia when they consumed less of the dietary pattern rich in vitamin B12, pantothenic acid, phosphorus, riboflavin, animal protein, total protein, vitamin B6, potassium, vitamin D, vitamin E, calcium, cholesterol, β-carotene, omega 3, magnesium, zinc, niacin, and selenium (NP1); or of those rich in iron, vegetable protein, thiamine, folate, total fiber, PUFA, insoluble fiber, vitamin A, vitamin K, alpha-tocopherol, copper, sodium, and retinol (NP2). The nutrients present in these patterns are characteristic of a diet that is rich in vegetables and milk and their derivatives, and they are related to bone metabolism, demonstrating the importance of nutrition with essential nutrients, especially calcium, phosphorus, magnesium, protein, and vitamin B12 for the maintenance of bone health. Further studies, especially longitudinal ones, are needed to confirm our results, especially with postmenopausal women, who may suffer more from the consequences of bone deterioration.

## Figures and Tables

**Table 1:** Distribution of sociodemographic, lifestyle, and clinical variables according to bone mineral density categories in postmenopausal women.

Variables	Total (*n* = 124)	BMD			*p* Value
		Normal (*n* = 27)	Osteopenia (*n* = 52)	Osteoporosis (*n* = 45)	
	**Mean ± SD**				
**Age (years)**	66.8 ± 6.1	64.2 ^a^ ± 5.3	66.6 ^a^ ± 6.0	68.6 ^b^ ± 6.1	**0.010**
**Time since menopause (years)**	19.6 ± 8.8	14.6 ^a^ ± 9.4	20.1 ^b^ ± 7.7	22.2 ^b^ ± 8.6	**0.002**
**BMI (kg/m^2^)**	27.3 ± 4.7	30.0 ^a^ ± 4.2	27.7 ^a^ ± 4.6	25.2 ^b^ ± 4.1	**<0.001**
	***n* (%)**				
**Age group (years) †**					**0.022**
50.0–59.9	13 (10.5)	5 (38.5)	7 (53.8)	1 (7.7)	
60.0–69.9	74 (59.7)	19 (25.7)	28 (37.8)	27 (36.5)	
≥70.0	37 (39.8)	3 (8.1)	17 (45.9)	17 (45.9)	
Color *					0.068
White	47 (37.9)	9 (19.1)	15 (31.9)	23 (48.9)	
Non-white	77 (62.1)	18 (23.4)	37 (48.1)	22 (38.6)	
Marital status *					0.079
No partner	60 (48.4)	8 (13.3)	29 (48.3)	23 (38.3)	
With partner	64 (51.6)	19 (29.7)	23 (35.9)	22 (34.4)	
**Education level †**					0.256
No schooling	11 (8.9)	-	7 (63.6)	4 (36.4)	
Elementary school	75 (60.5)	19 (25.3)	26 (34.7)	30 (40.0)	
High school	27 (21.8)	6 (22.2)	12 (44.4)	9 (7.3)	
University education	11 (8.9)	2 (18.2)	7 (63.6)	2 (18.2)	
**Employment status †**					0.616
Employed	21 (16.9)	6 (28.6)	9 (42.9)	6 (28.6)	
Unemployed	103 (83.1)	21 (20.4)	43 (41.7)	39 (37.9)	
Physical active level *					0.351
Insufficiently active	62 (50.0)	16 (25.8)	27 (43.5)	19 (30.6)	
Sufficiently active	62 (50.0)	11 (17.7)	25 (40.3)	26 (41.9)	
**Smoking †**					0.499
Smoker	6 (4.8)	-	3 (50.0)	3 (50.0)	
Non-smoker	118 (95.2)	27 (22.9)	49 (41.5)	42 (35.6)	
**Alcohol consumption †**					0.794
Consume	17 (13.7)	4 (23.5)	8 (47.1)	5 (29.4)	
Does not consume	107 (86.3)	23 (21.5)	44 (35.5)	40 (37.4)	
**Nutritional status †**					**<0.001**
Underweight	21 (16.9)	-	6 (28.6)	15 (71.4)	
Normal weight	53 (42.7)	9 (17.0)	23 (43.4)	21 (39.6)	
Overweight	18 (14.5)	7 (38.9)	7 (38.9)	4 (22.2)	
Obese	32 (25.8)	11 (34.4)	16 (50.0)	5 (15.2)	
Ca supplementation *					**<0.001**
Yes	81 (65.3)	9 (11.1)	35 (43.2)	37 (45.7)	
No	43 (34.7)	18 (41.9)	17 (39.5)	8 (18.6)	
Vit. D supplementation *					**<0.001**
Yes	71 (57.3)	7 (21.8)	31 (43.7)	33 (46.5)	
No	53 (42.7)	20 (37.7)	21 (39.6)	12 (22.6)	
**Antiresorptive drugs †**					**<0.001**
Yes	58 (46.8)	2 (3.4)	21 (36.2)	35 (60.3)	
No	66 (53.2)	25 (37.9)	31 (47.0)	10 (15.2)	

**†** Fisher’s Exact; *: Chi-squared. ANOVA, post hoc Tukey. Kruskal-Wallis, post hoc Bonferroni. SD: standard deviation. *p* values in bold: *p* < 0.05. Different superscript letters indicate a significant difference (*p* < 0.05) between groups. BMI: Bone mass index; BMD: Bone mineral density. Ca: Calcium; Vit. D: Vitamin D.

**Table 2:** Mean and standard deviation of energy and nutrient intake according to bone mineral density categories in postmenopausal women.

Nutrients	Total (*n* = 124)	BMD			*p* Value
		Normal (*n* = 27)	Osteopenia (*n* = 52)	Osteoporosis (*n* = 45)	
**Energy (kcal/d)**	2013.1 ± 791.4	2178.4 ± 729.3	1910.6 ± 818.3	2032.3 ± 794.3	0.357
**Carbohydrate (g/d)**	259.0 ± 40.0	276.2 ^a^ ± 45.8	241.5 ^b^ ± 34.7	268.8 ^a^ ± 34.6	**<0.001**
**Protein (g/d)**	87.6 ± 18.1	94.3 ^a^ ± 17.6	83.6 ^b^ ± 17.5	88.2 ^a,b^ ± 18.1	**0.040**
**Animal Protein (g/d)**	55.6 ± 19.9	59.3 ± 20.3	54.2 ± 20.0	55.2 ± 19.6	0.537
**Vegetal Protein (g/d)**	31.6 ± 9.9	34.5 ^a^ ± 10.9	29.0 ^b^ ± 7.1	32.9 ^a,b^ ± 8.7	0.014
**Total Fat (g/d)**	57.2 ± 12.6	66.9 ^a^± 15.2	53.2 ^b^ ± 10.6	55.9 ^b^ ± 10.1	<0.001
**Total Fibers (g/d)**	27.0 ± 8.0	27.6 ± 10.5	25.3 ± 6.2	28.5 ± 8.0	0.254
**Soluble Fibers (g/d)**	6.9 ± 2.1	6.7 ± 2.3	6.6 ± 2.0	7.3 ± 2.1	0.182
**Insoluble Fibers (g/d)**	19.7 ± 7.2	20.3 ± 10.2	18.3 ± 5.3	20.9 ± 6.9	0.249
**Cholesterol (g/d)**	257.6 ± 115.9	287.9 ± 126.5	253.0 ± 127.1	244.7 ± 92.9	0.304
**Saturated Fatty Acids (g/d)**	19.1 ± 5.9	22.5 ^a^ ± 7.4	17.3 ^b^ ± 4.8	19.2 ^b^ ± 5.3	**0.001**
**MUFA (g/d)**	17.6 ± 4.7	20.6 ^a^ ± 5.6	16.5 ^b^ ± 4.5	17.1 ^b^ ± 3.6	**0.001**
**PUFA(g/d)**	14.4 ± 3.4	16.6 ^a^ ± 3.1	13.7 ^b^ ± 2.7	14.0 ^b^ ± 3.8	**0.001**
**Trans Fatty Acids (g/d)**	1.3 ± 0.6	1.3 ± 0.5	1.2 ± 0.6	1.3 ± 0.6	0.453
**Vitamin A (UI/d)**	4903.0 ± 3617.0	5014.4 ± 2836.2	4509.9 ± 2634.2	5290.4 ± 4840.3	0.763
**Beta Carotene (µg/d)**	245.2 ± 141.5	274.7 ± 115.2	206.4 ± 115.2	272.4 ± 188.4	0.065
**Retinol (µg/d)**	1163.9 ± 635.9	1213.1 ± 529.9	1048.6 ± 458.6	1267.7 ± 832.1	0.314
**Vitamin D (µg/g)**	11.5 ± 10.4	16.0 ± 14.1	9.5 ± 7.4	11.1 ± 10.1	0.157
**Alpha Tocopherol (mg/d)**	8.7 ± 3.0	10.2 ^a^ ± 3.3	8.0 ^b^ ± 2.3	8.7 ^a,b^ ± 3.4	**0.008**
**Vitamin E (mg/d)**	7.2 ± 2.6	8.4 ^a^ ± 3.0	6.6 ^b^ ± 2.0	7.3 ^a,b^ ± 2.9	**0.023**
**Vitamin K (mcg/d)**	270.7 ± 258.4	282.6 ± 156.7	249.8 ± 172.7	287.7 ± 369.8	0.467
**Vitamin C (mg/d)**	184.3 ± 107.8	209.6 ± 107.0	162.6 ± 87.5	194.3 ± 125.6	0.103
**Thiamine (mg/d)**	1.8 ± 0.3	2.1 ^a^ ± 0.3	1.6 ^b^ ± 0.2	1.8 ^c^ ± 0.3	**<0.001**
**Riboflavin (mg/d)**	1.8 ± 0.4	2.1 ^a^ ± 0.4	1.7 ^b^ ± 0.3	1.9 ^a^ ± 0.5	**<0.001**
**Niacin (mg/d)**	24.1 ± 6.7	25.0 ± 6.5	23.3 ± 6.9	24.5 ± 6.6	0.520
**Pantothenic Acid (mg/d)**	6.2 ± 1.1	6.7 ^a^ ± 1.3	5.8 ^b^ ± 0.9	6.3 ^a,b^ ± 1.1	0.003
**Vitamin B6 (mg/d)**	2.3 ± 0.5	2.4 ± 0.6	2.2 ± 0.4	2.3 ± 0.4	0.106
**Folate (µg/d)**	509.8 ± 138.8	565.1 ^a^ ± 178.4	460.6 ^b^ ± 100.1	533.5 ^a^ ± 134.6	**0.010**
**Vitamin B12 (µg/d)**	3.4 ± 1.4	4.1 ^a^ ± 1.6	3.0 ^b^ ± 1.1	3.5 ^a,b^ ± 1.4	**0.004**
**Calcium (mg/d)**	742.4 ± 288.5	854.7 ^a^ ± 269.5	642.4 ^b^ ± 210.8	790.6 ^a^ ± 341.2	**0.003**
**Phosphorus (mg/d)**	1230.9 ± 249.3	1367.0 ^a^ ± 264.0	1134.0 ^b^ ± 188.5	1261.3 ^a^ ± 260.7	**<0.001**
**Magnesium (mg/d)**	329.3 ± 74.7	358.5 ± 92.0	306.7 ± 58.7	337.7 ± 73.3	**0.008**
**Iron (mg/d)**	12.6 ± 2.3	13.9 ^a^ ± 2.7	11.9 ^b^ ± 1.9	12.8 ^a,b^ ± 2.3	**0.001**
**Zinc (mg/d)**	10.5 ± 2.1	11.6 ^a^ ± 2.0	9.8 ^b^ ± 2.1	10.5 ^a,b^ ± 1.9	**<0.001**
**Copper (mg/d)**	1.5 ± 0.4	1.6 ± 0.6	1.4 ± 0.3	1.6 ± 0.5	0.145
**Selenium (µg/d)**	154.4 ± 88.7	189.6 ^a^ ± 104.6	149.0 ^b^ ± 89.7	139.4 ^b^ ± 72.0	**<0.001**
**Sodium (mg/d)**	2971.1 ± 596.7	3247.0 ^a^ ± 463.5	2777.7 ^b^ ± 441.3	3029.1 ^a,b^ ± 740.4	**0.002**
**Potassium (mg/d)**	3216.8 ± 667.4	3480.8 ^a^ ± 805.4	2969.1 ^b^ ± 805.4	3344.6 ^a^ ± 647.3	**0.001**
**Total Sugar (g/d)**	88.1 ± 30.6	96.4 ± 35.6	81.1 ± 28.8	91.1 ± 28.3	0.052
**Omega-3 (g/d)**	2.4 ± 1.1	3.0 ^a^ ± 1.4	2.2 ^b^ ± 0.8	2.3 ^b^ ± 1.1	**0.012**

ANOVA, post hoc: Tukey; Kruskal-Wallis, post hoc: Bonferroni. *p* values in bold: *p* < 0.05. Different superscript letters indicate a significant difference (*p* < 0.05) between groups. MUFA: Monounsaturated fatty acids; PUFA: Polyunsaturated fatty acids.

**Table 3:** Factor loadings of nutrients of the nutrient patterns identified in postmenopausal women.

Nutrients	Factor Loadings		
	NP1	NP2	NP3
**Vitamin B12**	**0.864**	−0.094	−0.169
**Pantothenic Acid**	**0.846**	0.162	0.274
**Phosphorus**	**0.837**	0.286	−0.052
**Riboflavin**	**0.757**	0.078	0.019
**Animal Protein**	**0.742**	−0.219	−0.346
**Total Protein**	**0.719**	0.179	−0.343
**Vitamin B6**	**0.683**	0.188	0.242
**Potassium**	**0.662**	0.332	−0.576
**Vitamin D**	**0.659**	0.227	0.095
**Vitamin E**	**0.568**	0.535	0.275
**Calcium**	**0.552**	−0.061	−0.041
**Cholesterol**	**0.537**	−0.161	−0.180
**β-Carotene**	**0.537**	−0.314	−0.071
**Omega 3**	**0.536**	0.524	−0.014
**Magnesium**	**0.437**	0.702	0.366
**Zinc**	**0.466**	0.268	−0.459
**Niacin**	**0.433**	0.122	−0.178
**Selenium**	**0.347**	0.295	−0.140
**Iron**	−0.128	**0.866**	−0.138
**Vegetal Protein**	−0.207	**0.864**	0.104
**Thiamine**	0.081	**0.798**	−0.149
**Folate**	0.026	**0.787**	0.289
**Insoluble Fibers**	0.048	**0.703**	0.392
**PUFA**	0.220	**0.664**	−0.417
**Total Fibers**	0.078	**0.681**	0.498
**Vitamin A**	0.117	**0.530**	0.285
**Vitamin K**	0.061	**0.611**	0.037
**Alpha-Tocopherol**	0.529	**0.606**	0.218
**Copper**	0.083	**0.580**	0.204
**Sodium**	−0.047	**0.438**	−0.287
**Retinol**	0.247	**0.470**	0.303
**Carbohydrate**	−0.353	0.200	**0.691**
**Total Sugar**	0.173	−0.239	**0.671**
**Soluble Fiber**	0.203	0.247	**0.539**
**Vitamin C**	0.357	0.159	**0.485**
**Total Fat**	0.385	0.015	**−0.765**
**MUFA**	0.363	−0.052	**−0.758**
**Saturated Fatty Acids**	0.321	−0.284	**−0.634**
**Trans Fatty Acids**	0.054	−0.159	**−0.520**
***Explicated Variance***	21.9%	20.4%	14.4%

NP1: Nutrient pattern 1; NP2: Nutrient Pattern 2; NP3: Nutrient Pattern 3. PUFA: Polyunsaturated fatty acids; MUFA: Monounsaturated fatty acids. Values in bold show the most characteristic nutrients of each nutrient pattern.

**Table 4:** Multivariate logistic regression between nutrient pattern 1 and nutrient pattern 2 tertiles, and bone mineral density categories of postmenopausal women.

	Osteopenia				Osteoporosis			
	Crude Model OR (CI 95%)	Model 1 OR (CI 95%)	Model 2 OR (CI 95%)	Model 3 OR (CI 95%)	Crude Model OR (CI 95%)	Model 1 OR (CI 95%)	Model 2 OR (CI 95%)	Model 3 OR (CI 95%)
**NP1**								
**1st T**	**6.00 (1.73–20.82)**	**6.66 (1.75–25.35)**	**6.65 (1.61–27.53)**	**6.64 (1.56–28.16)**	2.80 (0.81–9.74)	2.61 (0.62–10.90)	2.50 (0.45–13.74)	2.44 (0.43–13.78)
**2nd T**	**3.70 (1.15–11.86)**	**3.65 (1.05–12.64)**	**4.94 (1.31–18.55)**	**5.15 (1.32–20.07)**	2.29 (0.73–7.15)	1.78 (0.48–6.65)	3.34 (0.68–16.33)	3.48 (0.68–17.66)
**NP2**								
**1st T**	**4.84 (1.37–17.09)**	**5.06 (1.35–18.98)**	**4.99 (1.27–19.65)**	**5.03 (1.25–20.32)**	2.98 (0.87–10.16)	3.25 (0.81–13.00)	3.13 (0.63–15.65)	3.23 (0.63–16.67)
**2nd T**	**3.50 (1.13–10.84)**	**3.67 (1.10–12.18)**	3.54 (0.99–12.59)	3.59 (0.98–13.13)	1.31 (0.42–4.13)	1.40 (0.38–5.21)	1.24 (0.26–5.92)	1.22 (0.25–6.07)

Reference group: Third Tertile and BMD Normal; OR: Odds Ratio; CI 95%: Confidence Interval of 95%. Values in bold: *p* < 0.05. NP1: Nutrient Pattern 1; NP2: Nutrient Pattern 2; 1st T: First Tertile; 2nd T: Second Tertile. Model 1: adjusted for age, time since menopause, and BMI. Model 2 adjusted for age, time since menopause, BMI, physical active level, and antiresorptive drugs. Model 3: adjusted for age, time since menopause, BMI, physical active level, antiresorptive drugs, and calcium and vitamin D supplementation.

## Data Availability

Not applicable.

## Supplementary Materials

The following supporting information can be downloaded at: https://www.mdpi.com/article/10.3390/nu15071670/s1, Table S1: Distribution of means and frequency of sociodemographic, lifestyle, and clinical variables according to the nutrient patterns of postmenopausal women.
