# Supplementary material for: Nutrient Patterns and Risk of Osteopenia in Postmenopausal Women
Source: Nutrients. 2023 Mar 29;15(7):1670. doi: 10.3390/nu15071670 (PMC10096860; doi:10.3390/nu15071670)
Supplement: Supplementary file 1 [file nutrients-15-01670-s001.zip › nutrients-2254763-supplementary.pdf]

Table S1: Distribution of means and frequency of sociodemographic, lifestyle, and clinical variables according to the nutrient patterns of postmenopausal women.

VARIABLES	TERTILE OF NUTRIENT PATTERNS											
	NP 1			p value	NP 2			p value	NP 3			p value
	1st T	2nd T	3rd T		1st T	2nd T	3rd T		1st T	2nd T	3rd T	
	Mean ± SD											
**Age (Years)**	66.4 ± 6.4	67.6 ± 5.4	66.8 ± 6.1	0.586	67.6 ± 7.0	66.2 ± 5.0	66.7 ± 6.2	0.601	67.1 ± 6.0	65.8 ± 5.7	67.7 ± 6.6	0.364
**Time since menopause (Years)**	18.8 ± 8.2	20.2 ± 8.1	19.9 ± 10.2	0.766	20.7 ± 9.3	18.9 ± 7.2	19.3 ± 9.9	0.635	20.2 ± 8.6	17.9 ± 8.5	20.9 ± 9.3	0.265
**BMI (kg/m²)**	26.7 ± 4.3	26.6 ± 4.5	28.6 ± 5.0	0.091	27.2 ± 4.6	27.2 ± 4.7	27.4 ± 4.9	0.973	28.6 ± 4.9	26.9 ± 4.3	27.2 ± 4.7	0.082
	n (%)											
**Age group (Years)**				0.443				0.828				0.939
50.0 – 59.9	5 (38.5)	2 (15.4)	6 (46.2)		5 (38.5)	3 (23.1)	5 (38.5)		5 (38.5)	5 (38.5)	3 (23.1)	
60.0 – 69.9	26 (35.1)	24 (32.4)	24 (32.4)		22 (29.7)	27 (36.5)	25 (33.8)		25 (33.8)	24 (32.4)	25 (33.8)	
≥ 70.0	10 (27.0)	16 (43.2)	11 (29.7)		14 (37.8)	12 (32.4)	11 (29.7)		11 (29.7)	13 (35.1)	13 (35.1)	
**Color**				0.212				0.523				0.604
White	20 (42.6)	14 (29.8)	13 (27.7)		18 (38.3)	16 (34.0)	13 (27.7)		13 (27.7)	17 (36.2)	17 (36.2)	
Non-white	21 (27.3)	28 (36.4)	28 (36.4)		23 (29.9)	26 (33.8)	28 (36.4)		28 (36.4)	25 (32.5)	24 (31.2)	
**Marital status**				0.740				0.083				0.281
Without partner	18 (30.0)	22 (36.7)	20 (33.3)		23 (38.3)	23 (38.5)	14 (23.3)		24 (40.0)	18 (30.0)	18 (30.0)	
With partner	23 (35.9)	20 (31.3)	21 (33.1)		18 (28.1)	19 (29.7)	27 (42.2)		17 (26.6)	24 (37.5)	23 (35.9)	
**Education level**				0.531				0.079				0.619
No schooling	5 (45.5)	2 (18.2)	4 (36.4)		6 (54.5)	5 (45.5)	-		4 (36.4)	3 (27.3)	4 (36.4)	
Elementary school	26 (34.7)	28 (37.3)	21 (28.0)		25 (33.3)	22 (29.3)	28 (37.3)		28 (37.3)	23 (30.7)	24 (32.0)	
High school	7 (25.9)	10 (37.0)	10 (37.0)		9 (33.3)	9 (33.3)	9 (33.3)		6 (22.2)	10 (37.0)	11 (40.7)	

University school	3 (27.3)	2 (18.2)	6 (54.5)		1 (9.1)	6 (54.5)	4 (36.4)		3 (27.3)	6 (54.5)	2 (18.2)	
**Employement status**				0.998				0.115				0.533
Employed	7 (33.3)	7 (33.3)	7 (33.3)		9 (42.9)	3 (14.3)	9 (42.9)		5 (23.8)	9 (42.9)	7 (33.3)	
Unemployed	34 (33.0)	35 (34.0)	34 (33.0)		32 (31.1)	39 (37.9)	32 (31.1)		36 (35.0)	33 (32.0)	34 (33.0)	
**Physical active level**				0.354				0.844				**0.005**
Insuficiently active	21 (33.9)	24 (38.7)	17 (27.4)		21 (33.9)	22 (35.5)	19 (30.6)		24 (38.7)	26 (41.9)	12 (19.4)	
Suficiently active	20 (32.3)	18 (29.0)	24 (38.7)		20 (32.3)	20 (32.3)	22 (35.5)		17 (27.4)	16 (25.8)	29 (46.8)	
**Smoking**				0.870				0.123				0.529
Smoker	2 (33.3)	3 (50.0)	1 (16.7)		4 (66.7)	2 (33.3)	-		3 (50.0)	1 (16.7)	2 (33.3)	
Non-smoker	39 (33.1)	39 (33.1)	40 (33.9)		37 (31.4)	40 (33.9)	41 (34.7)		38 (32.2)	41 (34.7)	39 (33.1)	
**Alcohol consumption**				0.643				0.128				0.442
Consume	6 (35.3)	7 (41.2)	4 (23.5)		7 (41.2)	8 (47.1)	2 (11.8)		5 (29.4)	8 (47.1)	4 (23.5)	
Do not consume	35 (32.7)	35 (32.7)	37 (34.6)		34 (31.8)	34 (31.8)	39 (36.4)		36 (33.6)	34 (31.8)	37 (34.6)	
**Nutritional Status**				0.169				0.774				0.406
Underweigth	9 (42.9)	9 (42.9)	3 (14.3)		6 (28.6)	8 (38.1)	7 (33.3)		5 (23.8)	6 (28.6)	10 (47.6)	
Normal weigth	15 (28.3)	19 (35.8)	19 (35.8)		19 (35.8)	19 (35.8)	15 (28.3)		14 (26.4)	21 (39.6)	18 (34.0)	
Overweigth	9 (50.0)	5 (27.8)	4 (22.2)		5 (27.8)	4 (22.2)	9 (50.0)		8 (44.4)	5 (27.5)	5 (27.8)	
Obese	8 (25.0)	9 (28.1)	15 (46.9)		11 (34.4)	11 (34.4)	10 (31.3)		14 (43.8)	10 (31.3)	8 (25.0)	
**Ca supplementation**				0.640				0.765				0.640
Yes	29 (35.8)	27 (33.3)	25 (30.9)		28 (34.6)	28 (34.6)	25 (30.9)		25 (30.9)	27 (33.3)	29 (35.8)	
No	12 (27.9)	15 (34.9)	16 (37.2)		13 (34.6)	28 (34.6)	16 (37.2)		16 (37.2)	15 (34.9)	12 (27.9)	
**Vit. D supplementation**				0.716				0.339				0.587
Yes	25 (35.2)	22 (31.0)	24 (33.8)		27 (38.0)	21 (29.6)	23 (32.4)		23 (32.4)	22 (31.0)	26 (36.6)	

No	16 (30.2)	20 (37.7)	17 (32.1)		14 (26.4)	21 (39.6)	18 (34.0)		18 (34.0)	20 (37.7)	15 (28.3)	
**Antiresorptive drugs**				0.327				0.670				0.264
Yes	23 (39.7)	17 (29.3)	18 (31.0)		21 (36.2)	20 (34.5)	17 (29.3)		22 (37.9)	21 (36.2)	15 (25.9)	
No	18 (27.3)	25 (37.9)	23 (34.8)		20 (30.3)	22 (33.3)	24 (36.4)		19 (28.8)	21 (31.8)	26 (39.4)	
**BMD**				**0.038**				0.053				0.310
Normal	5 (18.5)	7 (25.9)	15 (55.6)		5 (18.5)	8 (29.6)	14 (51.9)		13 (48.1)	7 (25.9)	7 (25.9)	
Osteopenia	22 (42.3)	19 (36.5)	11 (21.2)		19 (36.5)	22 (42.3)	11 (21.2)		15 (28.8)	21 (40.4)	16 (30.8)	
Osteoporosis	14 (31.1)	16 (35.6)	15 (33.3)		17 (37.8)	12 (26.7)	16 (35.6)		13 (28.9)	14 (31.1)	18 (40.0)	

ANOVA, post-hoc: Tukey. Qui-Square Test; Fischer's Exact Test. P values in bold: $p < 0.05$. NP1: Nutrient Pattern 1; NP2: Nutrient Pattern 2; NP3: Nutrient Pattern 3; 1st T: First Tertile; 2nd T: Second Tertile; 3rd T: Third Tertile. SD: standard deviation. BMI: Body Mass Index; BMD: Bone Mineral Density. Ca: Calcium; Vit. D: Vitamin D.
